# Literary Note

**Published:** 1907-10

**Authors:** 


### LITERARY NOTE.

MESSRS. PARKE, DAVIS & Company, of Detroit, recently have issued a brochure describing and illustrating their biologic laboratories. This department of experimental medicine is nowhere excelled in its elaborateness or equipment for scientific research. The brochure gives one an imaginary tour through this portion of their great plant and is copiously illustrated with beautiful half-tones. It is printed on heavy calendered tinted paper, quarto in size, with illuminated type, and is about as handsome a specimen of the bookmaker's art as can be produced.

